# Effects of Tai Chi exercise on improving walking function and posture control in elderly patients with knee osteoarthritis: A systematic review and meta-analysis

**DOI:** 10.1097/MD.0000000000025655

**Published:** 2021-04-23

**Authors:** Yanwei You, Jianxiu Liu, Meihua Tang, Dizhi Wang, Xindong Ma

**Affiliations:** a Division of Sport Science and Physical Education, Tsinghua University, Beijing; b School of Kinesiology, Shanghai University of Sport, Shanghai, P.R. China.

**Keywords:** elderly patients, knee osteoarthritis, meta-analysis, posture control, Tai Chi, walking function

## Abstract

**Objective:**

It remains unclear whether Tai Chi is effective for walking function and posture control improvements in aged populations with knee osteoarthritis. The aim of this study was to systematically evaluate the effects of Tai Chi on improving walking function and posture control in elderly patients with knee osteoarthritis by updating the latest trial evidence.

**Methods:**

Web of Science, PubMed/Medline, Embase, Scopus, PEDro, and Cochrane library were searched up to October 1, 2020 to identify RCTs evaluating Tai Chi for improving walking function and posture control in older adults with knee osteoarthritis. The primary outcomes were walking function and posture control. Meta-analysis was performed with RevMan Version 5.3 software.

**Results:**

A total of 603 participants with knee osteoarthritis in the 11 trials were included. The results of meta-analysis showed that: The Tai Chi group was associated with better performance in 6-minute walk test (6 MWT), time up and go test (TUG) and “Western Ontario and McMaster Universities (WOMAC) Osteoarthritis Index” Physical Function Score than the control group ([MD: 46.67, 95% CI 36.91–56.43, *P* < .001]), ([MD: −0.89, 95% CI −1.16 to −0.61, *P* < .001]), ([MD: −11.28, 95% CI −13.33 to −9.24, *P* < .001]).

**Conclusion:**

This meta-analysis provided evidence from 11 RCTs that Tai Chi could be an excellent physical training strategy for improving walking function and posture control in older adults with knee osteoarthritis. Assuming that Tai Chi is at least effective and safe in most areas, it can be used as an adjuvant and reliable physical training strategy for walking function upgrading and balance control improvements for older patients with knee osteoarthritis.

**What is known**

- Tai Chi exercise may potentially improve balance and reduce falls among the older adults. When it comes to elderly patients with specific knee osteoarthritis, its curative effects for walking function and postural control ability remain controversial.

**What is new**

- The results of the meta-analysis with other new randomized controlled trials provide evidence support that Tai Chi Exercise has a positive effect on improving walking function and posture control in elderly patients with knee osteoarthritis. The present systematic review suggests that non-pharmaceutical therapies like Tai Chi could be used as an adjuvant and reliable physical training strategy for walking function upgrading and postural control improvements for older patients with knee osteoarthritis.

## 1 Introduction

Knee osteoarthritis (OA), a leading issue nowadays, is becoming one of the most common chronic degenerative joint disorders in the worldwide as the sharp increase of aging population. Converging epidemiological evidence demonstrates that knee OA is a growing prevalent joint disease, which is seen in group of different ages but it is more common among elder people.^[1,2]^ The characteristics of knee OA are the destruction of articular cartilage and bone changes of joint edge.^[3]^ Since the disease is often accompanied by joint deformity and function damage, walking dysfunction and postural instability caused by muscle weakness of lower extremities are common findings in these patients. As the consequences of aging, proprioception, and motor disturbances also result in postural imbalance and decreased walking function in this group of patients, which in part may lead to an elevated risk for falls during locomotion and then worse quality of life.

Walking and stabilizing posture are most basic abilities in human daily life. A recent publication has shown that stride length and pace are closely related to physical function and quality of life.^[4]^ Due to the functional limitation caused by knee OA, elderly patients may shorten their step length and slow down the travelling speed. In order to increase the stability of the knee joint, knee OA patients have no choice but to increase the simultaneous contraction of the lower extremity muscles at the expense of joint mobility, which may not only cause joint stiffness, but also soft tissue contracture.^[5,6]^ Explained from the theory of evolution and adaptation, on certain degree, this phenomenon is an adjustment of knee OA patients to the disease.

Over the past decades, a slice of assistive treatments for knee OA like acupuncture or kinesiology tape were used to alleviate pain, improve physical function, and minimize or slow the progression of disease.^[7,8]^ Currently, there is growing evidence showing that exercise and physiotherapy may also play an important role in improving the performance of postural control and walking related activities in patients with knee OA. Tai Chi is a traditional martial art widely practiced in China for centuries, which is also one of the non-pharmacological therapies recommended by the American College of Rheumatology for the rehabilitation method of knee OA.^[9]^

Some prior studies^[10,11]^ have reported that Tai Chi could potentially improve balance and reduce falls among the older adults. Many different type of researches focusing on Tai Chi's effects to improve walking function and posture control in elder knee OA groups used different dimensions of outcomes, which led to inconsistent and untrusted findings, so it was difficult to draw firm conclusions on the specific condition of knee osteoarthritis. Therefore, we conducted a meta-analysis of all randomized controlled trials (RCTs) with the same evaluation method to detect the effect of Tai Chi exercise on the walking function and posture control of the older adults with knee OA.

## 2 Methods

Ethical approval or patient consent was not required since the present study was a review of previous published researches.

### 2.1 Search strategy

The present meta-analysis was conducted on the basis of the PRISMA guidelines for systematic reviews and meta-analyses.^[12]^ The following databases were used for literature retrieval of potentially related articles from their inception through October 1, 2020: Web of Science, PubMed/Medline, Embase, Scopus, PEDro, and Cochrane library. In addition, manual citation searches of related original researches and review articles were conducted for any additional studies.

### 2.2 Study selection

The literature search was constructed by using Medical Subject Heading (MeSH) terms and Boolean operators. The search strategy for Web of Science were: “((‘Tai-ji’) OR (‘Taiji’) OR (‘Tai Chi’) OR (‘Tai Ji Quan’) OR (‘Chi,Tai’) OR (‘Taichi’) OR (‘Taijiquan’) OR (‘Tai Chi Chuan’) OR (‘Ji Quan,Tai’) OR (‘Quan,Tai Ji’) OR (‘Tai-yi’)) AND ((‘Knee Osteoarthritis’) OR (‘Knee OA’)) AND ((‘Walking’) OR (‘Posture’) OR (‘Balance’) OR (‘Function’) OR (‘Mobility’) OR (‘Physical Activity’) OR (‘Western Ontario and McMaster Universities Osteoarthritis Index’) OR (‘WOMAC’) OR (‘Time Up and Go Test’) OR (‘TUG’) OR (‘6-Minute Walk Test’) OR (‘6MWT’)),” and “((‘Randomized Controlled Trial’) OR (‘Randomized Clinical Trials’) OR (‘RCT’))” were also used to improve the search results from databases where MeSH terms were not used. Studies related to the influence of Tai Chi on walking function or posture control ability of patients with knee OA were included in this meta-analysis.

Clinical trial studies with regard to patients with Knee OA participated in Tai Chi exercises were included in our meta-analysis. We compared patients who had participated in Tai Chi to patients with attention control. The outcome measurement for walking function was the 6-minute walk test (6 MWT). In addition, we used time up and go test (TUG) and “Western Ontario and McMaster Universities Osteoarthritis Index” (WOMAC) Physical Function Score as the evaluating assessments for postural control ability.

### 2.3 Data extraction and quality assessment

Two reviewers independently extracted data on trial characteristics including: participants, interventions, duration time frequency, outcome assessments, and results. Disagreements were rechecked with a third reviewer and resolved by discussion. The data of overall information (first author's name, year of publication, region), characteristics of participants (sample size, age, gender, interventions, duration time) and the outcome assessments of the studies were extracted from each study.

The methodological quality and risk of bias in individual trials were assessed by Cochrane Collaboration's tool.^[13]^ The evaluation was performed according to the following risk of bias domains: selection bias (random sequence generation and allocation concealment), performance bias (blinding of participants and assessors), attrition bias (incomplete outcome data), reporting bias (selective reporting), and other bias. When the two reviewers showed different opinions, the third reviewer shall decide by discussion.

### 2.4 Statistical analysis

This meta-analysis was performed using Cochrane Collaboration review manager software (RevMan V.5.3). The mean difference (MD) and its 95% confidence interval (CI) were calculated to assess the effect of Tai Chi on improving walking function and posture control in elderly patients with knee osteoarthritis. A *P*-value of <.05 was considered as statistically significant.

The heterogeneity was evaluated by the Cochran's *Q*-test and the *I*^2^-index statistic, and a value for *P* < .05 accompanied by *I*^2^ > 50% indicated significant heterogeneity. If significant heterogeneity was detected, a random-effects model based on DerSimonian and Laird's method was used to calculate the overall effect size. If not, we used the fixed-effects model based on Mantel-Haenszel method.

## 3 Results

### 3.1 Study characteristics

**Figure 1:**
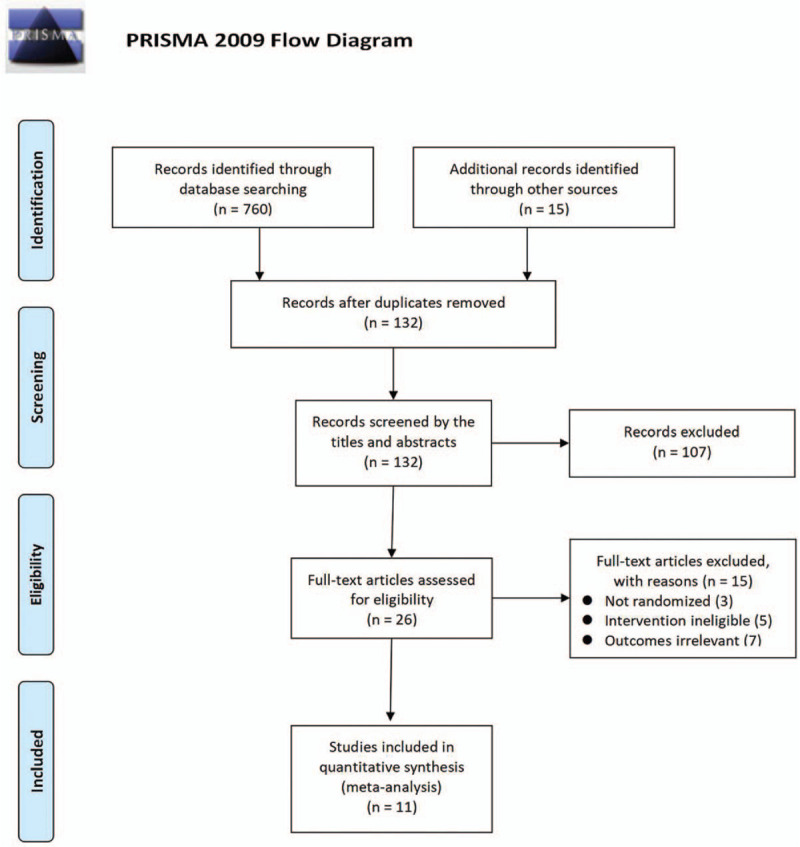
Flow chart of study selection and identification.

The flow chart of literature searches and study selections was clearly shown in Figure 1. A total of 775 citations were found in the electronic and additional search, of which 132 were excluded after deleting duplicates and filtering the titles and abstracts. The remaining 26 records were screened for full text and 11 studies including 603 participants were included finally.^[14–24]^

All included trials were RCTs. Table 1 presented the characteristics of included studies. The settings of the included studies were different, 5 studies were carried out in the United States,^[14,16,18–20]^ 4 in China,^[21–24]^ 1 in Australia, and 1 in Korea.^[15,17]^ The length of the intervention time ranged from 8 to 24 weeks. The types of Tai Chi included Yang-style^[14,16–18,20–24]^ and Sun-style.^[15,19]^

**Table 1:** Overview of the characteristics of the included studies.

First authors, year, country	Sample size	Age (range) or mean-SD (yr)	Sex, F/M	Duration weeks	Outcome assessments tools	Experimental group intervention	Control group intervention	Frequency (min or h/session per/wk)
Hartman (2000)^[14]^, USA	35	67 ± 8	30/5	12	TUG Test	Yang-style Tai Chi	Attention control	60 min/2 wk
Fransen (2007)^[15]^, Australia	97	70 ± 6.3	72/25	12	TUG Test/WOMAC Physical Function Score	Sun-style Tai Chi	Attention control	60 min/2 wk
Brismée (2007)^[16]^, USA	41	70 ± 9.2	34/7	12	WOMAC Physical Function Score	Yang-style Tai Chi	Attention control	40 min/3 wk
Wang (2009)^[18]^, USA	40	65 ± 7.8	30/10	12	6 MWT/WOMAC Physical Function Score	Yang-style Tai Chi	Attention control	60 min/2 wk
Lee (2009)^[17]^, Korea	44	69 ± 5	41/3	8	6 MWT/WOMAC Physical Function Score	Yang-style Tai Chi	Attention control	60 min/2 wk
Wortley (2013)^[20]^, USA	31	70 ± 5	22/9	10	6 MWT/TUG Test/WOMAC Physical Function Score	Yang-style Tai Chi	Attention control	60 min/3 wk
Tsai (2013)^[19]^, USA	55	78.9 ± 7.6	40/15	20	TUG Test	Sun-style Tai Chi	Attention control	20–40 min/3 wk
Zhu (2016)^[23]^, China	46	64.6 ± 3.4	46/0	24	WOMAC Physical Function Score	Yang-style Tai Chi	Attention control	60 min/2 wk
Lü (2017)^[21]^, China	46	64.6 ± 3.42	46/0	24	TUG Test	Yang-style Tai Chi	Attention control	60 min/3 wk
Li.L (2019)^[22]^, China	107	69.1 ± 3.9	57/50	12	6 MWT/WOMAC Physical Function Score	Yang-style Tai Chi	Attention control	45 min/5 wk
Li.J (2019)^[24]^, China	61	65.9 ± 5.8	23/38	16	6 MWT/TUG Test/WOMAC Physical Function Score	Yang-style Tai Chi	Attention control	60 min/3 wk

### 3.2 Methodological assessment of study quality

**Figure 2:**
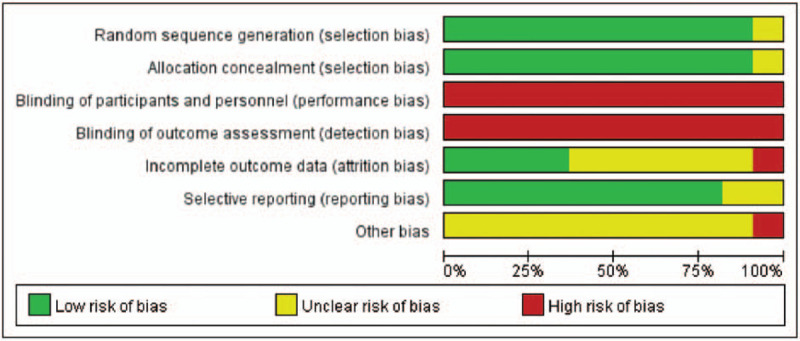
Risk of bias of the included studies (n = 11).

**Figure 3:**
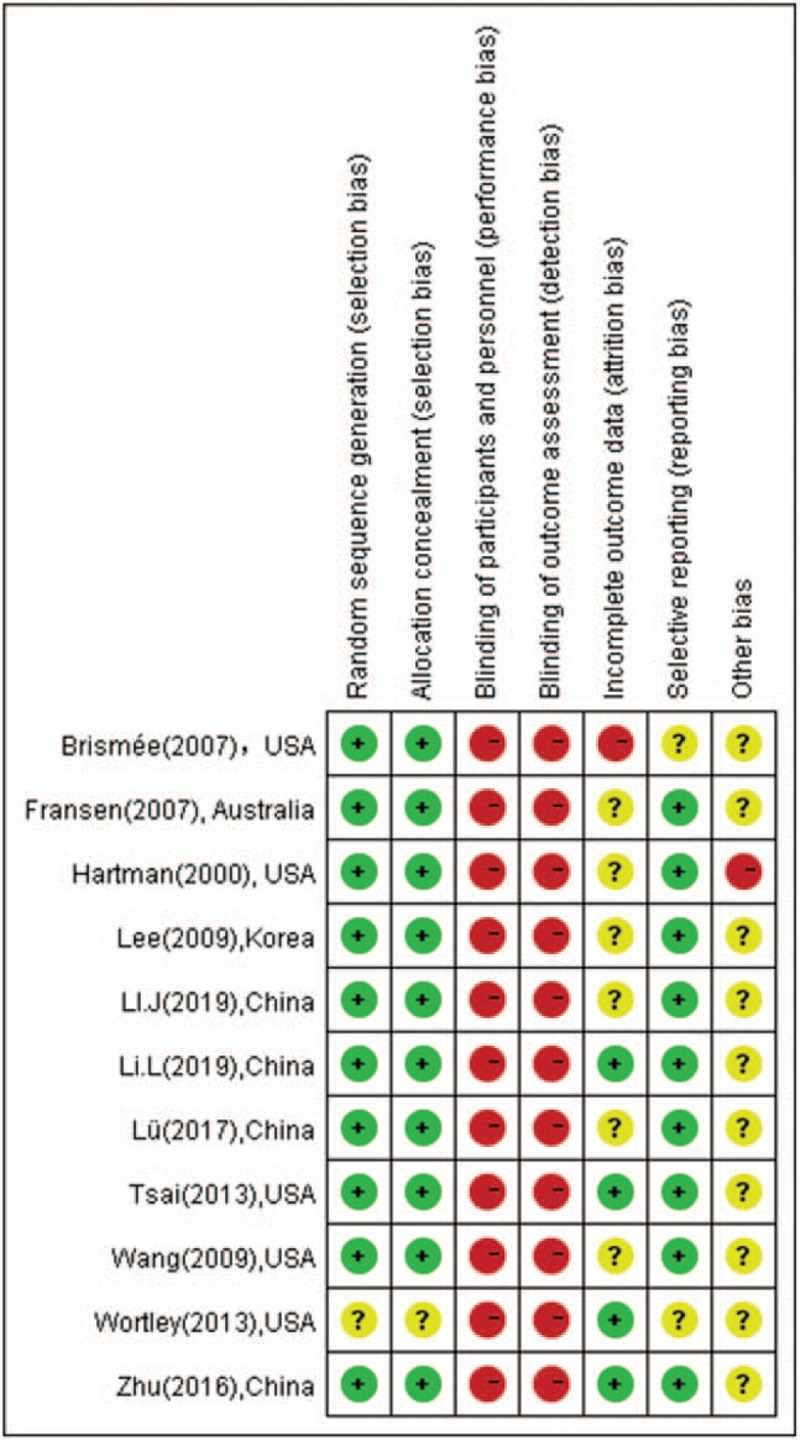
Summary of the risk of bias. The overall risk of bias, except for blinding (performance bias), was low.

Methodological quality assessment of the 11 included studies was summarized in Table 2, Figures 2 and 3. Ten trials showed methods of randomization by using a random number table or computer random method or the roll of a dice. Blinding of participants and assessors was not implemented in all studies. A total of 6 studies had unclear attrition bias. Selective reporting was found in 2 studies and other bias was difficult to assess.

**Table 2:** Methodological quality assessment of included studies.

			Blinding				
Studies	Random sequence generation (selection bias)	Allocation concealment (selection bias)	Participants, personnel	Assessors	Incomplete Outcome data (attrition bias)	Selective reporting (reporting bias)	Other bias
Hartman (2000)^[14]^, USA	L	L	H	H	U	L	U
Fransen (2007)^[15]^, Australia	L	L	H	H	U	L	U
Brismée (2007)^[16]^, USA	L	L	H	H	H	U	U
Wang (2009)^[18]^, USA	L	L	H	H	U	L	U
Lee (2009)^[17]^, Korea	L	L	H	H	U	L	U
Wortley (2013)^[20]^, USA	U	U	H	H	L	U	U
Tsai (2013)^[19]^, USA	L	L	H	H	L	L	U
Zhu (2016)^[23]^, China	L	L	H	H	L	L	U
Lü (2017)^[21]^, China	L	L	H	H	U	L	U
Li L (2019)^[22]^, China	L	L	H	H	L	L	U
Li J (2019)^[24]^, China	L	L	H	H	U	L	U

### 3.3 Results of meta-analysis

**Figure 4:**
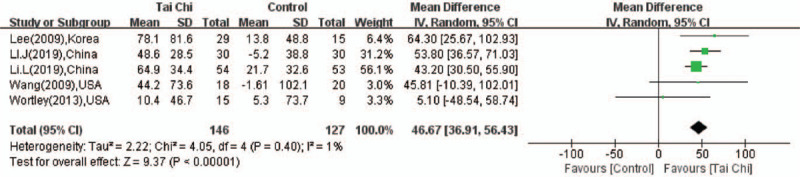
Forest plot showing the effect of Tai Chi on the 6 MWT in individuals with knee OA.

There were 5 studies^[17,18,20,22,24]^ in which the 6 MWT was performed. No significant heterogeneity (*P* = .4, *I*^2^ = 1%) was found among the five studies, so the fixed effect model was used to pool the data. The overall estimate of MD was 46.67 (95%CI: 36.91–56.43, *P* < .001; Fig. 4), indicating that the walking distance of the 6MWT of the older adults in the Tai Chi group was significantly enhanced compared with the control group.

**Figure 5:**
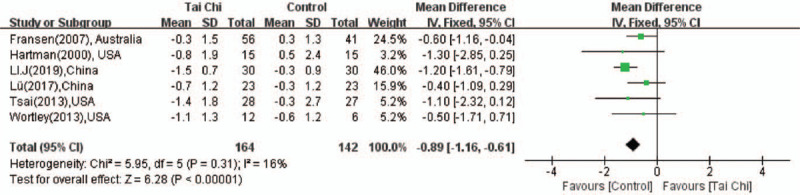
Forest plot showing the effect of Tai Chi on the TUG test in individuals with knee OA.

The results of TUG was reported in 6 articles.^[14,15,19–21,24]^ The significant heterogeneity was not existed in the studies (*P* = .31, *I*^2^ = 16%), so the fixed effect model was used. The pooled estimate (MD = −0.89; 95%CI: −1.16 to −0.61, *P* < .001; Fig. 5) demonstrated that the completion time of TUG test of elder people in the experiment group was definitely shortened compared with the control group.

**Figure 6:**
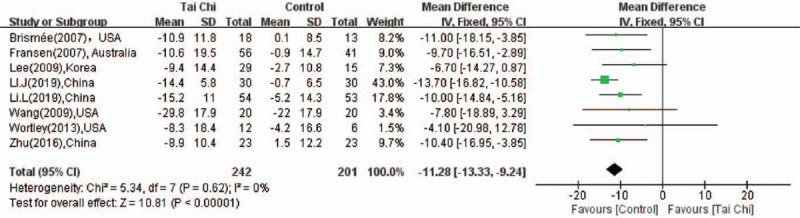
Forest plot showing the effect of Tai Chi on the WOMAC Physical Function Score in individuals with knee OA.

The WOMAC Physical Function Score was used in 8 studies.^[15–18,20,22–24]^ No significant heterogeneity (*P* = 0.62, *I*^2^ = 0%) was found among these studies, and fixed effect model was used. The pooled estimated of WOMAC Physical Function Score (MD = −11.28; 95%CI: −13.33 to −9.24, *P* < .001; Fig. 6) indicated that the postural control ability of the aged population in the experiment group was obviously increased compared with the control group.

## 4 Discussion

The purpose of the present review was to update and critically evaluate the effects of Tai Chi to improve walking function and posture control for older patients with knee osteoarthritis.

6 MWT was aimed to detect the walking function of subjects on smooth surface within 6 min by testing walking distance. We supposed that Tai Chi exercise could increase the stride length and frequency of older patients with knee OA by reducing the proportion of dual support phase in the gait period. In brief, the process of learning Tai Chi may have a positive impact on the neuromuscular control and brain activities of the older group, and then the enhancement of cognitive and concentration can help the elderly avoid external interference while walking.

TUG test is a dynamic balancing ability evaluation method for testing functional flexibility, which consists of several movement parts, including standing up from a chair, walking 3m, turning around, returning back to the chair, and then sitting down.^[25]^ This series of tasks have high requirements for posture control, because it not only requires coordinated body and strong muscle strength of lower extremities, but also needs good command of balance and movement control. Considering that in the process of practicing Tai Chi, the patient's hip, knee, and ankle joints are not maintained at a specific angle, but perform multi-directional rotation movements at different angles, alternating eccentric, and centripetal exercises. These series of exercises ultimately improve the patients’ physical control ability and improve their performance in TUG test.

When it comes to WOMAC Physical Function index, it has more elements to assess individuals’ comprehensive posture control ability, including 17 items such as using stairs, rising from sitting, standing, sitting, bending, etc. Symptom relief and functional gain can be observed from WOMAC Function Score reduction. Therefore, the alteration of two indexes can intuitively and significantly reflect the capacity changes of postural control. In our results, significant enhancement of WOMAC Physical Function Score and TUG Test verified that the ability of posture control has already been improved a lot under Tai Chi intervention.

To the best of our knowledge, this is the latest research to investigate Tai Chi exercise in relation to walking function and posture control improvement in elderly patients with knee OA. There were some similar reviews in previous publications.^[10,11,26–29]^ Compared with them, some novel findings and notable strengths of this study should be emphasized. The first one is that our findings can provide important insight into the benefits of Tai Chi in the setting of Knee OA and ultimately prove that non-pharmaceutical therapies like Tai Chi indeed plays an important role in enhancing quality of life in older adults with chronic knee symptoms. Secondly, we used systematic and consistent indicators to evaluate the effects of Tai Chi exercise on improving walking function and posture control in elderly patients with knee OA, in consideration that all studies included in this meta-analysis were RCTs, so the influence of confounding factors on the results was less. Thirdly, as an updated meta-analysis, a series of recently published studies were included, which enhanced the precision of the estimated effects and enabled us to broaden our horizons about Tai Chi exercise. In addition, considering the previous findings were likely to be affected due to publication bias and high heterogeneity, so the estimated preventive effect of Tai Chi might be overestimated, while in our research there was no significant heterogeneity among the included trials. Thus, our results were more reliable and our confidence in the findings was further increased by significant effect and stable analyses. Last but not least, this emerging research indicated not only encouraging evidence for Tai Chi exercise application but also pointed to the possibility of incorporating this program for prevention among those with significant risk factors for developing knee OA.

Some limitations of this study should be noted. First, the treatment protocol of Tai Chi has not yet been described, in most of the included studies the treatment duration and frequency were set at 60 min for each session, 2 to 3 sessions per week, for total of 8 to 24 weeks. Due to the limited sample size, it is still questionable whether different types of Tai Chi and long-term Tai Chi exercises can increase and expand the benefits. Secondary, similar to many other studies evaluating exercise intervention, blinding of participants, and assessors was hard to implement, which might lead to some quality assessment problems. In addition, the potential risk of bias may take place due to differences for the participants in the studies (such as region, gender, and age).

Noticeably, under the context of the coronavirus disease 2019 (COVID-19) pandemic,^[30]^ the isolation may cause less exercise and more health problems with knee osteoarthritis. Although outdoor games are typically more available, varied, and usually have more infrastructures, Tai Chi has its unique advantage as an exercise that can be performed and completed at home in such a special period. Moreover, Tai Chi exercise cannot only enhance joint range of motion, but also improve the blood circulation and respiratory function, which then help people boost the natural immunity to protect from the virus.

## 5 Conclusions

In conclusion, under the intervention of Tai Chi exercise, the walking function and posture control ability of elderly patients with knee OA were significantly improved. By using self-reported (WOMAC Physical Function Score) and experimental-reported (6 MWT and TUG test) outcomes, the benefits of Tai Chi for patients with knee OA disease were confirmed. Yang style and Sun style Tai Chi both contributed to great effects on improvement. Assuming that Tai Chi is at least effective and safe in most areas, it could be used as an adjuvant and reliable physical training strategy for walking function upgrading and balance control improvements. We are supposed to promote Tai Chi in the community in the future for its benefits of improving the dynamic stability and walking capacity of patients. However, since the mechanisms of improvement seem to be multifactorial, the neural mechanism of Tai Chi's effects on walking function and postural control is still worthy of further exploration and more high-quality RCTs are urgently needed to be supplemented to confirm these results.

## Author contributions

Yanwei You contributed to organize the study, prepare datasets, perform the statistical analysis, and write the manuscript. Jianxiu Liu participated in the design of the study and manuscript writing. Meihua Tang contributed to perform and collect the data. Dizhi Wang participated in the data preparation and analysis. Xindong Ma contributed to study organization and revision of the manuscript. All authors have read and approved the final manuscript, and agreed with the order of presentation of the authors.
